# Prevalence of TB and health-seeking behaviour

**DOI:** 10.5588/ijtld.22.0001

**Published:** 2022-05-01

**Authors:** N. Conan, E. Simons, L. Ohler, M. Mbatha, G. Van Cutsem, H. Huerga

**Affiliations:** 1 Epicentre, Paris, France; 2 Médecins Sans Frontières (MSF), Eshowe, South Africa; 3 Department of Health of South Africa, South Africa; 4 Southern Africa Medical Unit, MSF, Cape Town, South Africa; 5 Centre for Infectious Disease Epidemiology and Research, University of Cape Town, Cape Town, South Africa

Dear Editor,

South Africa remains among the countries with the highest incidence of TB (615 per 100,000 population in 2019).^1^ In 2017, a national TB prevalence survey reported an overall TB prevalence of 737/100,000, confirming the high TB burden;^2^ the HIV co-infection rate among TB cases was estimated at 28%.^2^ Here, we report on TB prevalence in the population of Eshowe/Mbongolwane in KwaZulu Natal, South Africa, and assess the proportion of individuals with undiagnosed TB and health-seeking behaviour for TB care among HIV-positive and HIV-negative people.

We conducted a cross-sectional, two-stage cluster sampling, population-based survey between August and December 2018. Clusters were selected with probability proportional to estimated population size. Within each cluster, 25 dwellings were randomly selected using spatial random sampling. All consenting people aged ≥15 years living in the selected dwellings were interviewed and asked about their demographic characteristics, TB treatment history, exposure to TB and health-seeking behaviour. All participants were tested for HIV at home. HIV pre- and post-test counselling was provided following South African recommendations.^3^ Participants not on TB treatment at the time of the interview were asked about the presence of the four WHO-recommended TB screening symptoms: cough, fever, night sweats and unexplained weight loss.^4^,^5^ Those with any of these symptoms were asked to provide two sputum samples for Xpert^®^ MTB/RIF (Cepheid, Sunnyvale, CA, USA) testing and Mycobacteria Growth Indicator Tube (MGIT) culture. Additional drug susceptibility testing was conducted if the MGIT culture was positive. Participants eligible for sputum collection were referred to the health facility of their choice for further TB investigations. Chest X-rays were not performed as part of the survey. Data analysis was performed using STATA v14 and 15 (StataCorp, College Station, TX, USA). Categorical variables were compared using Pearson χ^2^ or Fisher exact test, as appropriate. *P* < 0.05 was considered statistically significant. Ethical approval was received from the University of Cape Town Human Research Ethics Committee (Cape Town, South Africa; 320/2018), the Provincial Health Research Unit of the KwaZulu Natal Department of Health (Pietermaritzburg, South Africa; KZ_201807_26), and the Médecins Sans Frontières Ethics Review Board (ID1842). Prior to participation, participants provided written informed consent.

We included 4,182 participants of 5,130 eligible cases. The median age was 36 years (interquartile range [IQR] 22–56] and 67.8% were women; 941 (22.5%) participants were HIV-positive. Overall, 427 (10.2%) participants reported having been in close contact with a person diagnosed with TB in the previous 12 months, of whom 188 (44.0%) had sought medical care. Among the 941 HIV-positive participants, 831 (88.3%) reported that they had sought HIV care in the past, of whom 358 (43.1%) had received TB preventive therapy. Of the 4,182 participants, 368 (8.8%) had at least one episode of TB prior to the survey (23.5% among HIV-positive vs. 4.5% among HIV-negative; *P* < 0.001). The median number of TB treatments received prior to the study was 1 (IQR 1–1). In total, 214/221 (96.8%) HIV-positive participants with a previous episode of TB were aware of their HIV-positive status compared to 634/720 (88.1%) of those without previous TB episodes (*P* < 0.001). Of the 368 participants who had at least one episode of TB prior to the survey, 367 had received TB treatment. Of these, 12 (3.3%) reported stopping treatment because they started to feel better (*n* = 4), due to cost burden and lack of support (*n* =3), because they were recommended by a professional to discontinue treatment (*n* = 3) or as there was no improvement of health condition (*n* =2). Among the 354 participants who reported completing treatment, 92 (26%) received drugs for more than 6 months and among these, 19 (20.7%) received injectable drugs. Seven (1.9%) of the 368 participants who experienced at least one prior episode of TB, also presented a TB episode at the time of the survey.

Of the 4,154 participants who were not on TB treatment at the time of the survey, 304 (7.3%) were eligible for sputum collection, and 205/304 (67.4%) were tested using Xpert and culture. Among these 205 participants, 4 (2.0%) tested positive (Figure). Including the 26 individuals on TB treatment at the time of the survey, a total of 30 (0.7%) participants had TB: 15/941 (1.6%) among HIV-positive participants and 15/3,221 (0.5%) among HIV-negative participants (*P* < 0.001). Of these, 13.3% (4/30) were undiagnosed prior to the survey: 1/15 (6.7%) among HIV-positive participants and 3/15 (20.0%) among HIV-negative participants. Based on these results, the estimated prevalence of TB in the surveyed area was 717/100,000 (95% confidence interval [CI] 485–1,023) and 821 individuals (95% CI 555–1,171) had TB, of which 109 (95% CI 74–156) were undiagnosed.

**Figure:**
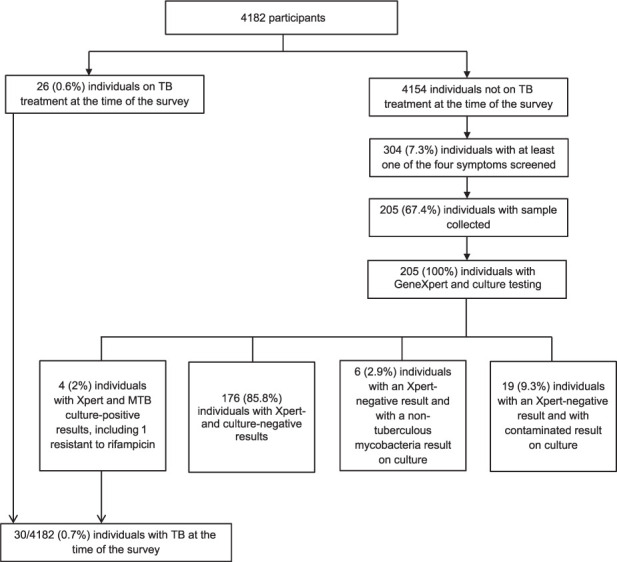
Flow chart of TB testing results, KwaZulu-Natal, South Africa, 2018.

TB prevalence in the Eshowe and Mbongolwane sub-districts of KwaZulu-Natal, particularly among HIV-positive individuals, was very high, in line with the 2017 National TB Survey.^2^ Similar surveys conducted in Ghana^6^ and Zambia^7^ have found lower prevalence, at respectively 356/100,000 (288–425) and 638/100,000 (502–774). However, in our study, half of the participants who had TB at the time of the survey were HIV-positive, which is higher than the 28% found in the 2017 National Survey.^2^ Most of the patients with active TB at the time of the survey were already receiving TB treatment. The proportion of undiagnosed TB participants at the time of survey was high among HIV-negative individuals, but lower in HIV-positive cases. However, the proportion of undiagnosed TB participants, as well as the TB prevalence, may have been underestimated, as participants with asymptomatic or subclinical TB may have been missed during the survey. For example, a recent Zambian study revealed that 12.5% of confirmed TB cases were either asymptomatic or did not present the four WHO-recommended TB screening symptoms.^8^ Similar results were found in Cambodia,^9^ where approximately 40% of individuals with a positive Xpert result were asymptomatic. Moreover, a lower proportion of participants had a positive TB symptom screening, compared to the National Survey (7.3% vs. 25.8%).^2^ Previous studies in South Africa have shown that a substantial proportion of household contacts diagnosed with TB were asymptomatic,^10^,^11^ which may explain why less than half of close TB contacts in our study had sought medical care. We also found that, despite current recommendations,^1^,^5^ less than half of the HIV-positive participants under HIV care had received TB preventive therapy, as reported elsewhere.^1^,^12^

Our study had some limitations. First, chest X-ray was not performed as part of the TB screening strategy, which may have led to an underestimation of TB prevalence and the proportion of undiagnosed participants. In addition, some individuals were not able to provide a sputum sample; forms of pulmonary and extrapulmonary TB may, as a result, have been missed. Finally, self-reporting bias may also have occurred, with possible underestimation of TB history and treatment.

In conclusion, these results show that TB diagnosis should be enhanced for the people living in Eshowe/Mbongolwane. In addition, TB contacts tracing and investigation and TB preventive therapy for HIV-positive individuals should be reinforced in this area.
